# Cavitary Pulmonary Tuberculosis in an Infant

**DOI:** 10.7759/cureus.74678

**Published:** 2024-11-28

**Authors:** Sofia Branco, Sara Nogueira Machado, Inês Azevedo, Isabel Carvalho, Sónia Silva

**Affiliations:** 1 Pediatrics, Unidade Local de Saúde Póvoa de Varzim/Vila do Conde, Póvoa de Varzim, PRT; 2 Pediatrics, Unidade Local de Saúde São João, Porto, PRT; 3 Pediatrics, Unidade Local de Saúde do Alto Ave, Guimarães, PRT; 4 Gynecology-Obstetrics and Pediatrics, University of Porto, Porto, PRT; 5 Epidemiology Research Unit, Public Health Institute, University of Porto, Porto, PRT; 6 Childhood Tuberculosis Department, Centro de Diagnóstico Pneumológico de Gaia, Porto, PRT; 7 Pediatrics, Unidade Local de Saúde Vila Nova de Gaia e Espinho, Porto, PRT

**Keywords:** atypical presentation, cavitation, infant, mycobacterium tuberculosis, tuberculosis

## Abstract

Tuberculosis (TB) continues to pose a significant health challenge globally and in Portugal. Diagnostic challenges persist, especially in infants, where TB often presents with atypical symptoms.

A previously healthy three-month-old male infant from Vila Nova de Famalicão, Portugal, was admitted with cough, rhinorrhea, respiratory distress, and high-grade fever. After chest radiography and blood tests, he was treated with ampicillin for community-acquired pneumonia (CAP). Initial antibiotic therapy proved ineffective, and subsequent imaging showed necrotizing pneumonia. Thoracic computed tomography and bronchoscopy revealed a significant consolidation with cavitation and lymphatic involvement, as well as extrinsic compression of the right main bronchus. Gastric aspirates (GA) and bronchoalveolar lavage (BAL) washings grew *Mycobacterium tuberculosis*. Treatment eventually with antituberculosis drugs and prednisolone resulted in clinical and radiological improvement. Subsequent immunological evaluations were normal.

## Introduction

According to Portuguese national surveillance data, the incidence of tuberculosis (TB) has fallen below the threshold defined as low incidence (<20/100,000 population) since 2014 [1]. In 2016, Portugal adopted targeted Bacillus Calmette-Guérin (BCG) vaccination plans, focusing exclusively on children within specific risk groups, in alignment with WHO recommendations for countries with low incidence rates [2]. However, TB cases in Portugal continue to experience a prolonged time to diagnosis within the European context and among high-income countries. Delayed diagnoses are largely due to a lack of consideration for TB, limited experience among healthcare professionals, and the underestimation of symptoms [3].

In 2022, an estimated 1.25 million children worldwide developed TB, accounting for 12% of the global TB incidence burden. However, 51% of these cases were not diagnosed or not reported to national TB programs [4,5]. This detection gap is particularly pronounced among infants, where TB poses unique diagnostic challenges due to atypical presentations, non-specific signs and symptoms, the paucibacillary nature of the disease, and the difficulty in accessing a suitable sample for diagnosis in this age group [6-9]. Unlike older children, infants are at higher risk of rapid progression from TB infection to TB disease, manifesting as pulmonary or extrapulmonary TB. Their immature immune systems predispose them to developing severe and disseminated forms of the disease, with increased morbidity and mortality [7-10]. 

This case report of severe pulmonary TB with cavitation in a Portuguese infant with no known immunodeficiency illustrates that TB still needs to be considered in cases with community-acquired pneumonia (CAP).

## Case presentation

A previously healthy three-month-old male infant from Vila Nova de Famalicão, Portugal, was admitted to the Pediatric Emergency Department due to cough and rhinorrhea lasting for four days, along with high-grade fever for three days. His father had rhinorrhea in the preceding two weeks, and there was no personal or family history of travel. The infant had received all vaccinations included in the Portuguese National Vaccination Program except for the Bacillus Calmette- Guérin (BCG) vaccination, despite a history of TB in close family members from the maternal lineage, specifically the grandmother, grandfather, and uncle, with the last active case successfully treated three years ago.

On admission, the infant was febrile (rectal temperature of 39.9ºC) and presented nasal obstruction, tachypnea with accessory muscle use, oxygen saturation of 98% in room air, and normal breathing sounds. The cardiac and neurological examinations were normal, and no palpable enlargement of the liver or spleen was noted. The infant had not refused feedings and had satisfactory weight gain.

Blood analysis (Table 1) revealed leukocytosis with neutrophilia (white blood cell count 21790/µL, neutrophils 13728/µL, lymphocytes 6057/µL), an elevated C-reactive protein (CRP) level of 100.5 mg/L and a procalcitonin level of 0.29 ng/mL. Serum electrolytes, renal function, and liver enzymes were within normal parameters.

**Table 1:** Laboratory results N/A - not applicable

Test	Result	Unit	Reference range
Hemoglobin	10.1	g/dL	9.5 – 13.5
White blood cells	21.79	10³/µL	6.0 – 17.5
Neutrophils	13.728	10³/µL	1.0 – 8.5
Lymphocytes	6.057	10³/µL	4.0 – 13.5
Platelets	654	10³/µL	150 – 400
Glucose	105	mg/dL	60 - 110
Urea	15	mg/dL	10 – 20
Creatinine	0.4	mg/dL	0.2 – 0.5
Sodium	136	mmol/L	135 – 145
Potassium	4.7	mmol/L	3.5 – 5.0
Chloride	105	mmol/L	100 – 110
Aspartate aminotransferase	23	U/L	10 – 40
Alanine aminotransferase	15	U/L	10 – 40
C-reactive protein	100.5	mg/L	0 – 5
Procalcitonin	0.29	ng/mL	0.00 – 0.05
Blood cultures	No growth	N/A	N/A

**Figure 1:**
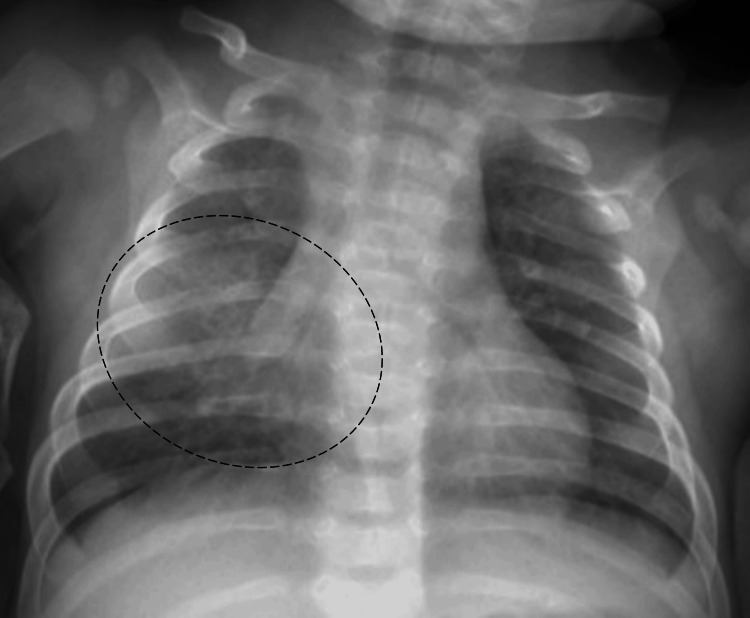
Chest radiograph at admission Anteroposterior chest radiograph showing a right opacity, resulting in an obliteration of the cardiac silhouette (circle).

His chest radiograph revealed a right pulmonary opacity (Figure 1), consistent with pneumonia. Empiric intravenous ampicillin was initiated.

During hospitalization, the infant continued to experience persistent fever and progressive worsening of respiratory distress. A repeat chest radiograph revealed no significant changes. Ampicillin was replaced with ceftriaxone. However, the infant's inflammatory markers remained elevated, with a maximum CRP level of 146.8mg/L, the respiratory virus polymerase chain reaction panel was negative, and the blood cultures showed no growth for five days. On day six, a thoracic computed tomography (CT) scan suggested the possibility of necrotizing pneumonia. Clindamycin and vancomycin were added to the treatment regimen. Due to persistent fever and respiratory symptoms, an interferon-gamma release assay (IGRA) was performed and yielded a negative white cell response. 

**Figure 2:**
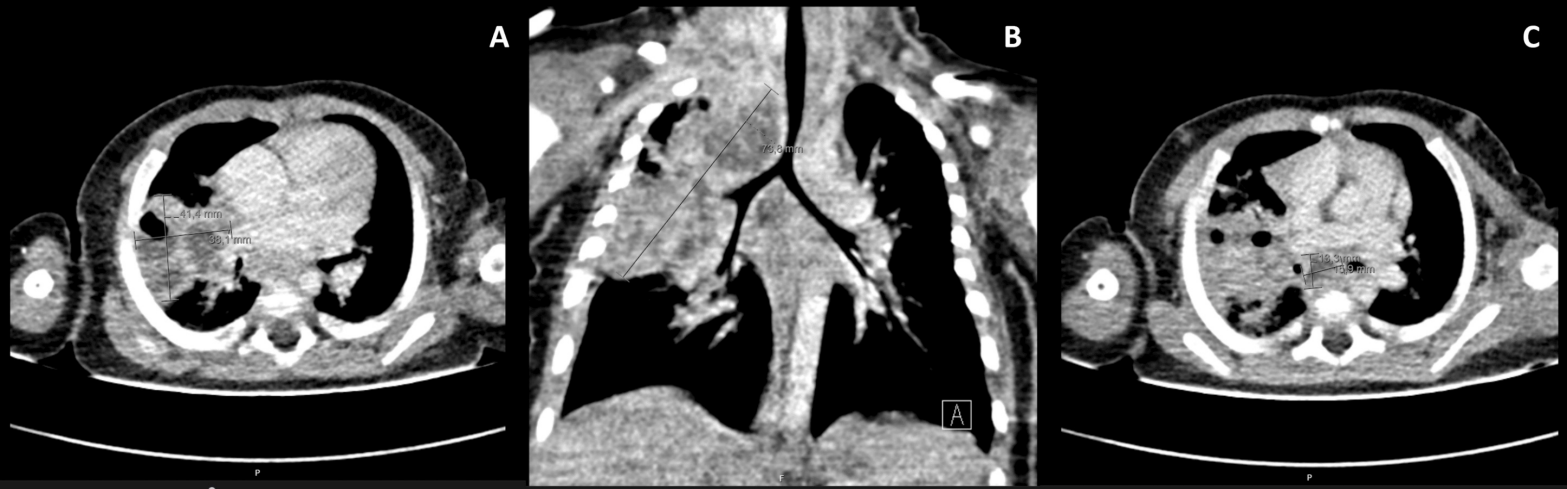
Contrast-enhanced thoracic computed tomography Contrast-enhanced thoracic computed tomography scan revealing in the upper right lobe a voluminous consolidation measuring 74x41x38mm (A, B) with areas of cavitation and contiguous with necrotic lymph nodes in the right hilar and mediastinal regions, the largest located in the subcarinal area measuring 16x13mm (C).

Subsequently, the infant was transferred to the Pediatric Pulmonology Unit of a tertiary hospital, where a repeat contrast-enhanced CT scan revealed a voluminous consolidation measuring 74x41x38 mm, with areas of cavitation and contiguous with necrotic lymph nodes in the right hilar and mediastinal regions, the largest located in the subcarinal area measuring 16x13mm (Figure 2). 

These extensive necrotic lung, pleural, and lymph node infiltrates suggested the possibility of granulomatous infection, such as TB or a bacterial/fungal infection. Bronchoscopy revealed an extrinsic compression of the right main bronchus without any endobronchial lesions. The infant received prednisolone. The acid-fast bacilli smear from GA and bronchoalveolar lavage (BAL) were both positive and nucleic acid amplification tests for the Mycobacterium tuberculosis complex yielded positive results in GA, BAL, and stool specimens. Ceftriaxone, clindamycin, and vancomycin were discontinued, and isoniazid, rifampicin, pyrazinamide, and ethambutol (HRZE) were initiated. Cultures on BAL and GA fluids grew *M. tuberculosis* susceptible to all first-line TB drugs, with negative Genotype® MTBDRsl assay (Bruker-Hain Diagnostics, Nehren, Germany). Additionally, routine bacteriological and mycological cultures on BAL washings were negative.

The infant boy was successfully treated for pulmonary TB. Extrapulmonary manifestations beyond intrathoracic lymph node involvement were not seen.

The infant improved clinically and was discharged after 14 days of oral corticosteroid and HRZE therapy, to continue treatment at an outpatient TB center, with follow-up at the pulmonology and immunodeficiency disorders outpatient clinic. Subsequent screening of family members and close contacts revealed that the grandmother had an active TB infection, identifying her as the likely infectious source.

**Figure 3:**
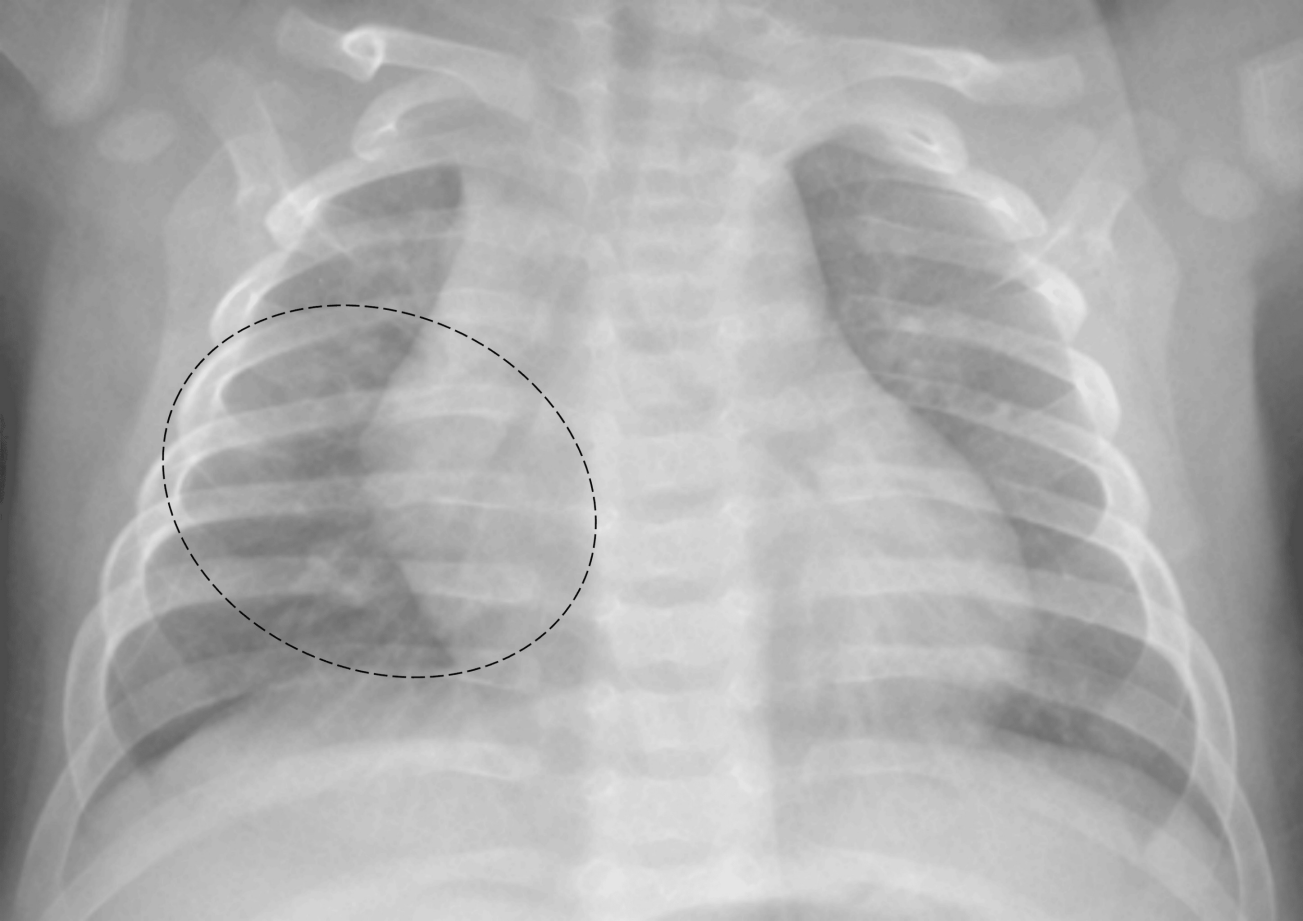
Chest radiograph at two months Anteroposterior chest radiograph at a two-month follow-up, showing a marked improvement (circle)

At follow-up, substantial radiological improvement was observed (Figure 3). The infant remained asymptomatic, demonstrating normal growth and development. Prednisolone was continued for a total of four weeks, followed by a gradual tapering over an additional four weeks. Antituberculosis treatment, consisting of a two-month intensive phase with four drugs of HRZE followed by a continuation phase of HR (isoniazid and rifampicin) for six months, was successfully completed without adverse effects. Immunology studies, including human immunodeficiency virus serology, IL-12/IFN γ axis, NADPH oxidase gp91phox expression, and oxidative burst, yielded normal results.

## Discussion

This case underscores the importance of considering TB as a differential diagnosis in infants presenting with prolonged fever and respiratory symptoms, particularly when no identifiable infectious etiology is evident, and the clinical course is deteriorating. TB in infants rapidly progresses to fulminant lung and mediastinal disease following exposure and often leads to delayed recognition of TB as a potential cause of acute pneumonia in this age group. In this infant clinical case, his acute clinical presentation of CAP and later complicated CAP, combined with the initial absence of known contact with an active TB case, contributed to TB being overlooked.

In infants, TB typically presents with mediastinal and hilar lymphadenopathy and parenchymal lung changes. The occurrence of expansile caseating pneumonia is uncommon [6,11,12]. In our case, the compression and obstruction of the right bronchus by necrotic lymph nodes in the upper lobe, followed by endobronchial spread, resulted in dense lobar consolidation with cavitations.

It is important to note that immunological tests for detecting TB, such as the IGRA and the tuberculin skin test, have limited utility in infants due to reduced sensitivity [13]. Our infant boy with cavitary pulmonary TB and mediastinitis had a false-negative IGRA result. Accurate diagnosis of TB in pediatric patients often necessitates the collection of biological samples, including sputum, stool, urine, blood, and gastric washings, to test for the presence of the M. tuberculosis pathogen. In cases of pulmonary TB among infants, samples such as GA or BAL are particularly crucial. The culture of *M. tuberculosis* remains the gold standard for the diagnosis of TB [9]. Molecular tests, such as the Genotype® MTBDRsl assay, excluded resistance to second-line TB drugs [14].

The presence of TB in a close family member, in this case, the grandmother, underscores the necessity of comprehensive contact tracing and screening, including outside family and close contacts. Even in low-incidence countries, control of TB requires public health officials to identify potential sources of infection and prevent further transmission within the community. In spite of meeting the eligibility criteria for BCG vaccination [2], the infant here was not immunized. While BCG is commonly administered to prevent TB meningitis and disseminated disease, its effectiveness in preventing pulmonary TB remains uncertain [15].

## Conclusions

This case of necrotizing pneumonia with cavitation and mediastinitis due to TB emphasizes the necessity of maintaining a high index of suspicion for TB in infants, even in those without obvious epidemiological risk factors. Early recognition, prompt diagnosis, and appropriate management are essential for achieving favorable outcomes and preventing disease transmission within the community. 
